# CD40 ligand stimulation affects the number and memory phenotypes of human peripheral CD8^+^ T cells

**DOI:** 10.1186/s12865-023-00547-2

**Published:** 2023-06-30

**Authors:** Haeyoun Choi, Hyun-Joo Lee, Hyun-Jung Sohn, Tai-Gyu Kim

**Affiliations:** 1 Department of Microbiology, College of Medicine, The Catholic University of Korea, 222 Banpo-daero, Seoul, 06591 Republic of Korea; 2 Catholic Hematopoietic Stem Cell Bank, College of Medicine, The Catholic University of Korea, 222 Banpo-daero, Seoul, 06591 Republic of Korea

**Keywords:** CD40L, CD8^+^ T cell, Proliferation, Memory, Artificial antigen presenting cell

## Abstract

**Background:**

CD40L is primarily expressed on activated CD4^+^ T cells and binds to CD40 which is expressed by various cells including dendritic cells, macrophages and B lymphocytes. While CD40-CD40L interaction is known to be direct between B cells and CD4^+^ T cells which results in proliferation and immunoglobulin isotype switching, antigen presenting cells (APCs) were thought to be involved in the delivery of CD4^+^ help to CD8^+^ T cells by cross-talk between CD4^+^ and CD8^+^ T cells and APCs. However, subsequent study demonstrated that CD40L signal can be directly delivered to CD8^+^ T cells by CD40 expression on CD8^+^ T cells. Since most studies have been carried out in murine models, we aimed to investigate the direct effect of CD40L on human peripheral CD8^+^ T cells.

**Results:**

Human peripheral CD8^+^ T cells were isolated to exclude the indirect effect of B cells or dendritic cells. Upon activation, CD40 expression on CD8^+^ T cells was transiently induced and stimulation with artificial APCs expressing CD40L (aAPC-CD40L) increased the number of total and central memory CD8^+^ T cells and also pp65 specific CD8^+^ T cells. Stimulation with aAPC-CD40L also resulted in higher proportion of central memory CD8^+^ T cells.

**Conclusions:**

Our study suggests that CD40L has an effect on the increased number of CD8^+^ T cells through CD40 expressed on activated CD8^+^ T cells and has influence on memory CD8^+^ T cell generation. Our results may provide a new perspective of the effect of CD40L on human peripheral CD8^+^ T cells, which differ according to the memory differentiation status of CD8^+^ T cells.

**Supplementary Information:**

The online version contains supplementary material available at 10.1186/s12865-023-00547-2.

## Background

CD40L, a 33 kDa glycoprotein and a member of the tumor necrosis factor (TNF) gene family is primarily expressed on activated T cells, especially activated CD4^+^ T cells. It binds to CD40 which is expressed by various cells including dendritic cells, macrophages and B lymphocytes [1, 2]. The role of CD40-CD40L on B cells has been extensively studied and CD40-CD40L signaling results in B cell activation, proliferation and immunoglobulin isotype switching, leading to effective immune response [3, 4].

In addition to the effect of CD40L on B cells, several studies demonstrated the important role of CD40L in CD8^+^ T cell priming and memory generation [5–9]. While CD40-CD40L interaction is known to be direct between B cells and CD4^+^ T cells, antigen presenting cells (APCs) were thought to be involved in the delivery of CD4^+^ help to CD8^+^ T cells by cross-talk between CD4^+^ and CD8^+^ T cells and APCs [7, 8, 10, 11]. It has been assumed that when CD4^+^ T cells recognize the antigen loaded on MHC class II presented by APC, B7 expressed on APC stimulate CD4^+^ T cells to express CD40L. The CD40L bind to CD40 on APCs and these “conditioned” APCs in turn drive CD8^+^ T cell activation and memory generation. Several studies demonstrated the important role of CD40L in CD8^+^ T cell priming and memory generation. Specifically, absence of CD40L stimulation resulted in poor CTL responses and CD4^+^ T cell help could be replaced by monoclonal antibody against CD40 in generating OVA-specific CTLs [7, 8]. However, subsequent study demonstrated that CD40L signal can be directly delivered to CD8^+^ T cells by showing that CD8^+^ T cell memory generation is possible with CD4^+^ T cell help even when the APCs are CD40^−/−^ [9]. Most studies have been carried out in murine models and the effect of CD40L on human peripheral CD8^+^ T cells further needs to be elucidated.

In this study, we investigated the direct effect of CD40L on human peripheral CD8^+^ T cells by using artificial antigen presenting cells (aAPCs) expressing CD40L [12]. Pure CD8^+^ T cells were isolated using flow cytometry as CD3 and CD8 double positive population to exclude the indirect effect of B cells or dendritic cells. After sorting, the purity was over 98% and each subset of memory CD8^+^ T cells depending on the expression of CD45RO and CD62L was used to evaluate the effect of CD40L according to the differentiation status of CD8^+^ T cells. To investigate the effect of CD40L in the context of antigen-specific responses, we used pp65 antigen of human cytomegalovirus (HCMV). Our results suggest that CD40L affects proliferation of CD8^+^ T cells and influence on the memory phenotype of CD8^+^ T cells.

## Results

### CD40 expression is induced on CD8^+^ T cell after stimulation

**Fig. 1:**
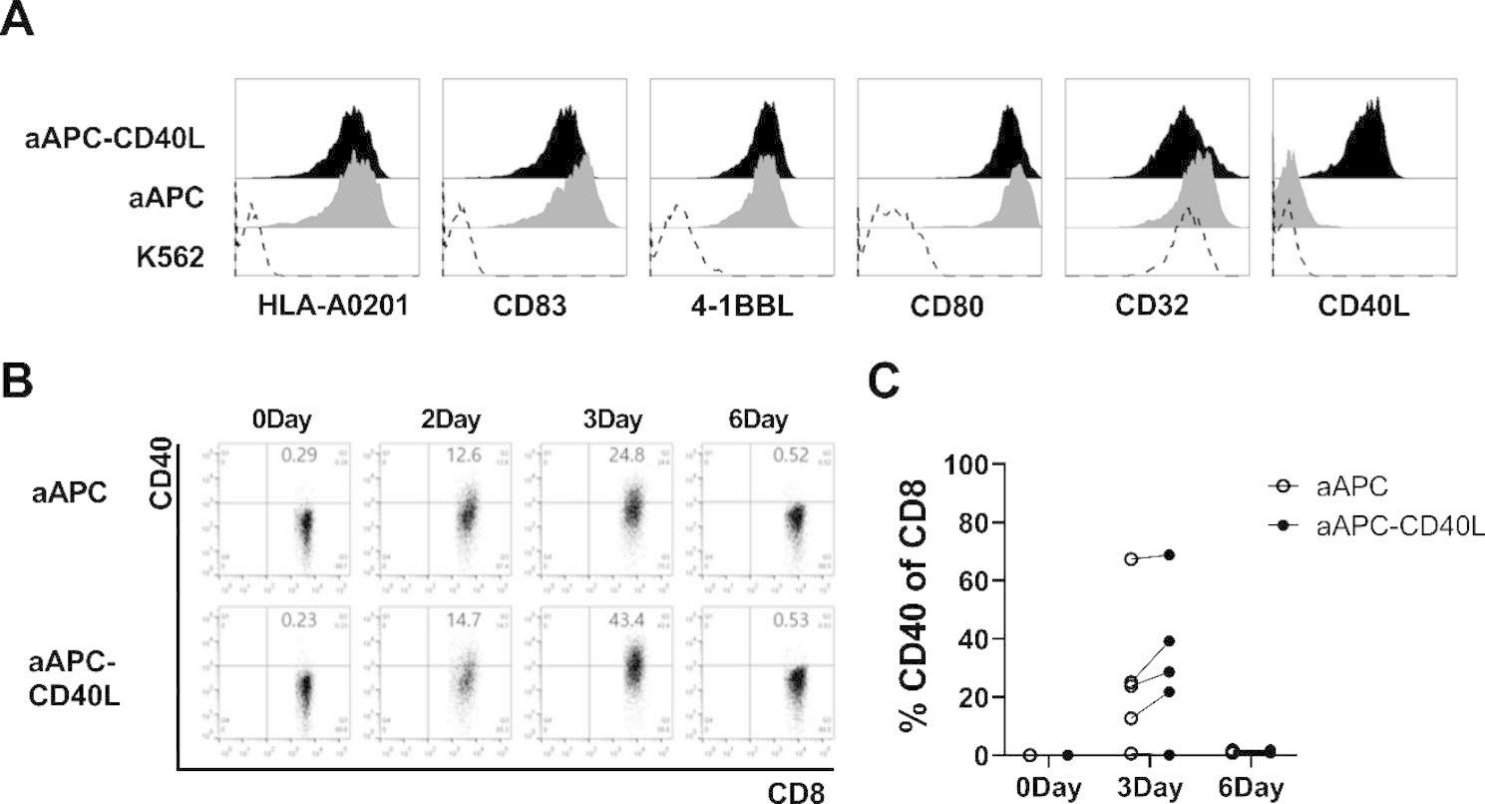
CD8^+^ T cells transiently express CD40 after stimulation. (**A**) Establishment of aAPC expressing single HLA, co-stimulatory molecules and/or CD40L. K562 cells were transduced with recombinant lentiviral plasmids encoding HLA-A*02:01, CD83, 4-1BBL, CD80, CD32 (aAPC) and lentiviral plasmid encoding CD40L was additionally transduced to establish aAPC-CD40L. Expression levels of the co-stimulatory molecules on K562, aAPC and aAPC-CD40L were analyzed by flow cytometry. (**B, C**) CD40 expression on human peripheral blood CD8^+^ T cells after stimulation with aAPC or aAPC-CD40L. CD8^+^ T cells were isolated from five healthy donor PBMCs by magnetic sorting and were stimulated with anti-CD3 loaded aAPC/aAPC-CD40L in the presence of IL-2 (10U/mL). Expression of CD40 on CD8^+^ T cells were analyzed on 0, 3 and 6 days after stimulation by flow cytometry. Representative FACS data and data from 5 donors on day 0, 3 and 6 are shown as dot plot

To investigate the direct effect of CD40L on CD8^+^ T cells, K562 based aAPC expressing various co-stimulatory molecules was established. Lentivirus vector encoding CD83, 4-1BBL, CD80, CD32 and HLA-A*0201 were transduced to establish aAPC and CD40L was additionally expressed to further establish aAPC-CD40L. aAPC and aAPC-CD40L expressed high levels of transduced molecules compared to K562 cells (Fig. 1A).

Next, CD40 expression level on CD8^+^ T cells were analyzed (Fig. 1B, C). On day 0, CD40 expression on CD8^+^ T cells were minimal. However, 3 days after stimulation with anti-CD3 loaded aAPC or aAPC-CD40L, CD40 expression on aAPC or aAPC-CD40L stimulated CD8^+^ T cells transiently increased to 25.9% ± 25.2 and 31.7% ± 25.2 (mean ± standard deviation) respectively. On day 6, CD40 expression decreased to baseline expression (1.3% ± 0.4 and 1.2% ± 0.4). CD40 expression did not statistically differ between aAPC stimulated CD8^+^ T cells and aAPC-CD40L stimulated CD8^+^ T cells. In addition, transient increase in CD40L expression on aAPC or aAPC-CD40L stimulated CD8^+^ T cells was seen on day 3 (3.0% ± 3.8 and 6.7% ± 8.3) and decreased to baseline expression on day 6 (Additional File Fig. 1).

These results show that CD40 expression on CD8^+^ T cells could be induced transiently after stimulation. However, CD40L expressed on aAPC itself may not be the important factor but other non-specific stimulation of CD8^+^ T cells might lead to the upregulation of CD40 expression on CD8^+^ T cells.

### Stimulation of CD40L to CD40 results in the increased number of CD8^+^ T cells

**Fig. 2:**
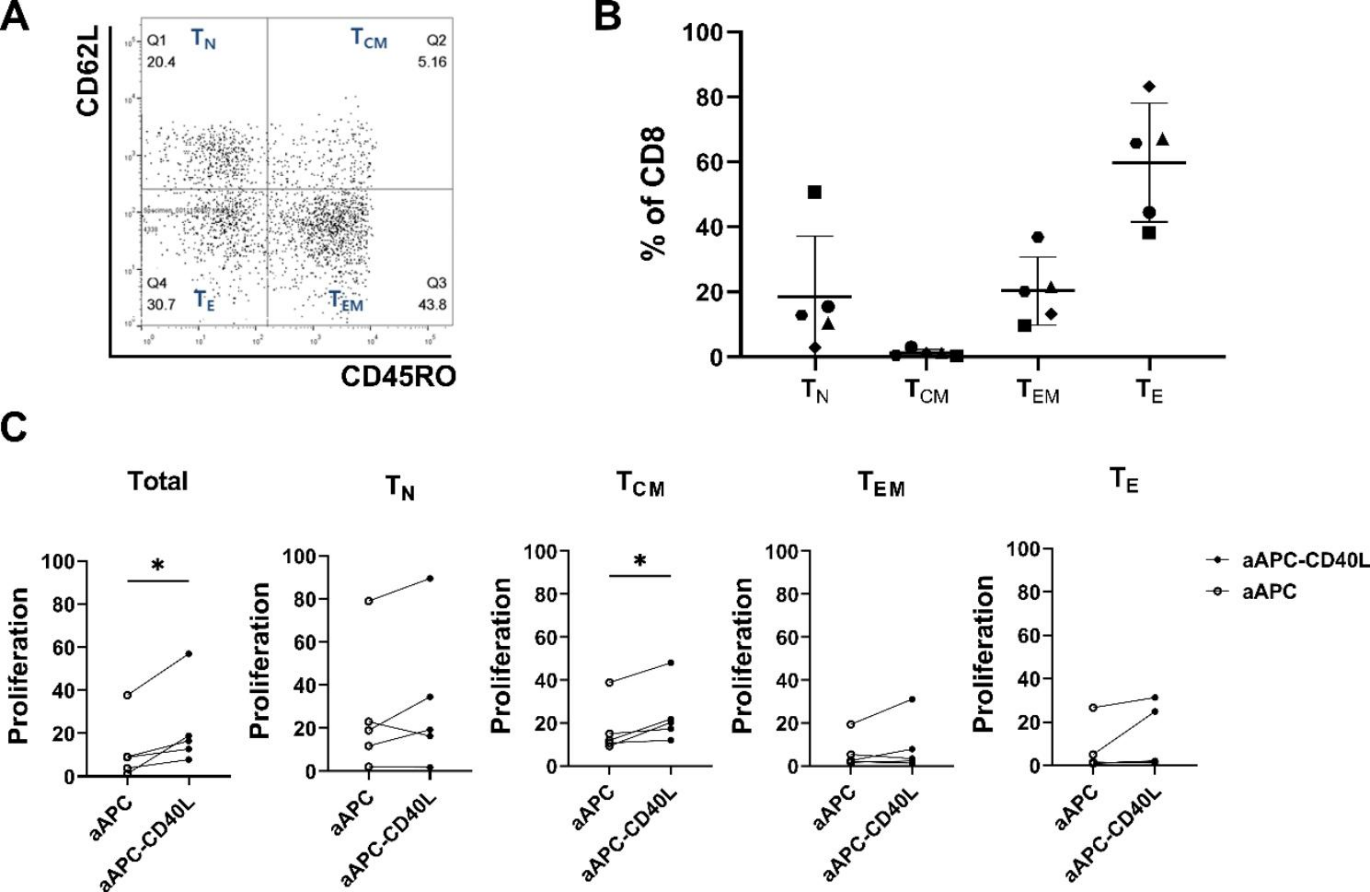
CD40L expressed on aAPC enhances the proliferation of CD8^+^ T cells stimulated by anti-CD3 antibody. CD8^+^ T cells from five healthy donors were sorted into memory subsets based on CD45RO and CD62L expression: naïve (CD45RO^−^/CD62L^+^), central memory (CD45RO^+^/CD62L^+^), effector memory (CD45RO^+^/CD62L^−^) and effector CD8^+^ T cells (CD45RO^−^/CD62L^−^). Unsorted total CD8^+^ T cells and sorted CD8^+^ T cell memory subsets were weekly stimulated with irradiated anti-CD3 loaded aAPC or aAPC-CD40L in the presence of IL-2(10U/mL). On day 12, fold expansion of CD8^+^ T cells was analyzed. (**A**) Representative data of CD45RO and CD62L expression on CD8^+^ T cells. (**B**) Proportion of each memory subsets of CD8^+^ T cells are shown (n = 5). Bar and error indicate mean and SD. (**C**) Fold expansions of CD8^+^ T cells stimulated with aAPC were compared to those of CD8^+^ T cells stimulated with aAPC-CD40L on day 12 (n = 5). Wilcoxon paired rank test one tailed, *p < 0.05

Human peripheral blood CD8^+^ T cells consists of those with diverse memory differentiation status. To accurately analyze the effect of CD40L on each memory subset of CD8^+^ T cells, CD8^+^ T cells were sorted according to the expression levels of CD45RO and CD62L (Fig. 2A). Analysis of the memory subsets of CD8^+^ T cells revealed that the most prevalent memory subset of CD8^+^ T cells in human peripheral blood was effector(T_E_) subset, followed by effector memory(T_EM_), naïve(T_N_), and central memory(T_CM_) (T_E_ ; 59.8% ± 18.3%, T_EM_ ; 20.3% ± 10.5%, T_N_ ; 18.5% ± 18.6% and T_CM_ ; 1.3% ± 1.1%) (Fig. 2B).

After sorting on day 0, unsorted total CD8^+^ T cells and each subset of sorted memory subset of CD8^+^ T cells were weekly stimulated with irradiated, anti-CD3 loaded aAPC or aAPC-CD40L. After two rounds of stimulation, the fold expansion of total and each subset of CD8^+^ T cells was analyzed by dividing the CD8^+^ cell number on day 12 by the number on day 0 (Fig. 2C). Fold expansion of aAPC-CD40L stimulated CD8^+^ T cells were significantly higher than that of aAPC stimulated CD8^+^ T cells in total CD8^+^ T cells and in T_CM_ subset (12.2 ± 14.7 versus 22.5 ± 19.7 and, 17.2 ± 12.3 versus 23.9 ± 14.0 respectively, P < 0.05).

Because CD40 expressing dendritic cells and B cells were depleted and the experiment was carried on sorted CD8^+^ T cells, these results indicate that CD40L stimulation results in the increased number of CD8^+^ T cells, and the increase of T_CM_ subset mostly attributes to the increase of total CD8^+^ T cell number.

### Effect of CD40L stimulation on the memory phenotype of CD8^+^ T cells

**Fig. 3:**
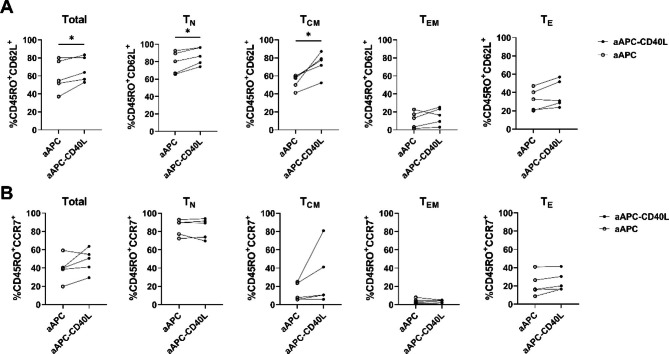
Effect of CD40L stimulation on the memory generation of CD8^+^ T cells. (**A, B**) Total or sorted memory subsets of CD8^+^ T cells were weekly stimulated with irradiated anti-CD3 loaded aAPC or aAPC-CD40L in the presence of IL-2(10U/mL). CD45RO^+^CD62L^+^ proportion and CD45RO^+^CCR7^+^ proportion of CD8^+^ T cells stimulated with aAPC were compared to those of CD8^+^ T cells stimulated with aAPC-CD40L on day 12 (n = 5). Wilcoxon paired rank test one tailed, *p < 0.05

To clarify the effect of CD40L on the memory phenotype of each memory subset of CD8^+^ T cells, the expression of CD45RO, CD62L, and CCR7 on aAPC or aAPC-CD40L stimulated CD8^+^ T cell subsets were analyzed on day 12. The percentage of CD45RO^+^CD62L^+^ population was significantly higher in aAPC-CD40L stimulated total CD8^+^ T cells compared to that of aAPC stimulated total CD8^+^ T cells (67.3% ± 13.8 versus 59.9% ± 17.9, respectively). Also, significantly higher CD45RO^+^CD62L^+^ population in aAPC-CD40L stimulated group was seen in T_N_ and T_CM_ subsets (T_N_; 86.4% ± 10.0 versus 78.9% ± 12.5, T_CM_ ; 73.6% ± 13.1 versus 53.6% ± 8.0 respectively) (P < 0.05) (Fig. 3A). When CD45RO and CCR7 expressions were assessed, no significant difference of CD45RO^+^CCR7^+^ cell population was shown between aAPC stimulated CD8^+^ T cells and aAPC-CD40L stimulated CD8^+^ T cells (Fig. 3B). These data demonstrate the effect of CD40L on the central memory phenotype of CD8^+^ T cells especially in naïve and T_CM_ subsets.

### CD40L stimulation results in the HCMV pp65-specific CD8^+^ T cell proliferation

**Fig. 4:**
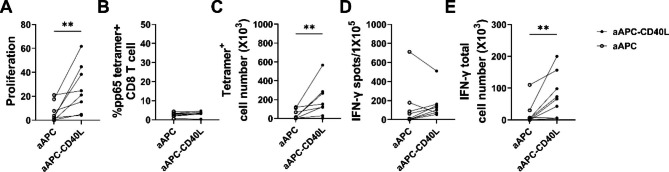
CD40L expressed on aAPC enhances the proliferation of CD8^+^ T cells stimulated with CMV pp65 antigen. Sorted CD8^+^ T cells were weekly stimulated with irradiated aAPC or aAPC-CD40L loaded with HLA-A*02:01 restricted peptides of pp65 in the presence of IL-2(10U/mL). After two rounds of stimulation, fold expansion, flow cytometry analyses and ELISPOT assay was done on day 12. (**A**) Fold expansion of CD8^+^ T cells stimulated with antigen-loaded aAPC was compared to that of CD8^+^ T cells stimulated with antigen-loaded aAPC-CD40L. (**B, C**) Tetramer positive CD8^+^ T cell proportion and tetramer positive CD8^+^ T cell number was compared between aAPC stimulated group and aAPC-CD40L stimulated group. Tetramer positive CD8^+^ T cell number was calculated as (% of tetramer positive CD8^+^ T cell)×(total CD8^+^ T cell number on day 12). (**D, E**) For ELISPOT assay, CD8^+^ T cells were co-cultured with antigen-loaded T2 cells. After 24 h of incubation, ELISPOT was done to analyzed the IFN-γ secretion from CD8^+^ T cells. IFN-γ spots were compared between aAPC stimulated CD8^+^ T cells and aAPC-CD40L stimulated CD8^+^ T cells. IFN-γ secreting total cell number was analyzed by multiplying the IFN-γ spot number to the number of CD8^+^ T cells on day 12 (n = 8). Wilcoxon paired rank test one tailed, **p < 0.005

Next, the effect of CD40L on the number of antigen-specific CD8^+^ T cells were analyzed. To address this, total CD8^+^ T cells were weekly stimulated with aAPC or aAPC-CD40L loaded with HLA-A*02:01 restricted peptides of pp65. After two rounds of stimulation, fold expansion of CD8^+^ T cells were analyzed on day 12. Expansion of pp65 loaded aAPC-CD40L stimulated group was 4 fold higher compared to pp65 loaded aAPC stimulated counterpart (26.8 ± 20.1 versus 6.8 ± 8.1, P < 0.005) (Fig. 4A). In addition to the effect shown on the increased number of anti-CD3 stimulated CD8^+^ T cells, CD40L also had a significant effect on the number of pp65 stimulated CD8^+^ T cells.

To analyze the frequency of pp65-specific CD8^+^ T cells among proliferated CD8^+^ T cells, tetramer positive populations were analyzed on day 12. No significant difference in the frequency of pp65 tetramer positive CD8^+^ T cell was shown in aAPC-CD40L stimulated group compared to aAPC stimulated group (Fig. 4B). To compare the tetramer positive CD8^+^ T cell number between aAPC stimulated group and aAPC-CD40L stimulated group, tetramer positive CD8^+^ T cell number was calculated by multiplying the tetramer positive population by the total CD8^+^ T cell number on day 12. Although the frequency of tetramer positive CD8^+^ T cells between aAPC-CD40L stimulated pp65-specific CD8^+^ T cells and aAPC stimulated counterparts were similar, significant increase in total yield of aAPC-CD40L stimulated pp65-specific CD8^+^ T cell led to statistically significant increase in total tetramer positive CD8^+^ T cell number (40.9 ± 50.4 versus 193.2 ± 180.7; aAPC versus aAPC-CD40L, respectively, P < 0.005) (Fig. 4C).

Furthermore, the effect of CD40L on the cell number of IFN- γ secreting pp65-specific CD8^+^ T cells were analyzed. After stimulation with pp65 peptide pulsed T2 cells, ELISPOT was done to analyze the IFN-γ secretion from CD8^+^ T cells on day 12. There was no significant difference in the IFN-γ spot number between antigen specific CD8^+^ T cells stimulated with aAPC and that of antigen specific CD8^+^ T cells stimulated with aAPC-CD40L. Specifically, the number of IFN-γ spots in pp65 specific CD8^+^ T cells was 132.3 ± 242.0 in aAPC stimulated CTLs compared with 156.1 ± 148.1 in aAPC-CD40L stimulated CTLs (Fig. 4D). However, when total IFN-γ secreting CD8^+^ T cell numbers were calculated by multiplying IFN-γ spots by total CD8^+^ T cell number, total pp65 specific IFN-γ secreting CD8^+^ T cell number was 4 fold higher with statistical significance in aAPC-CD40L stimulated group compared to aAPC stimulated counterpart (80.9 ± 68.9 versus 19.6 ± 37.9, P > 0.05) (Fig. 4E). As in tetramer positive CD8^+^ T cell number, significantly higher number in aAPC-CD40L stimulated antigen-specific CD8^+^ T cell led to the significant increase in IFN-γ secreting CD8^+^ T cell number.

### Effect of CD40L stimulation on the memory generation of pp65-specific CD8^+^ T cells

**Fig. 5:**
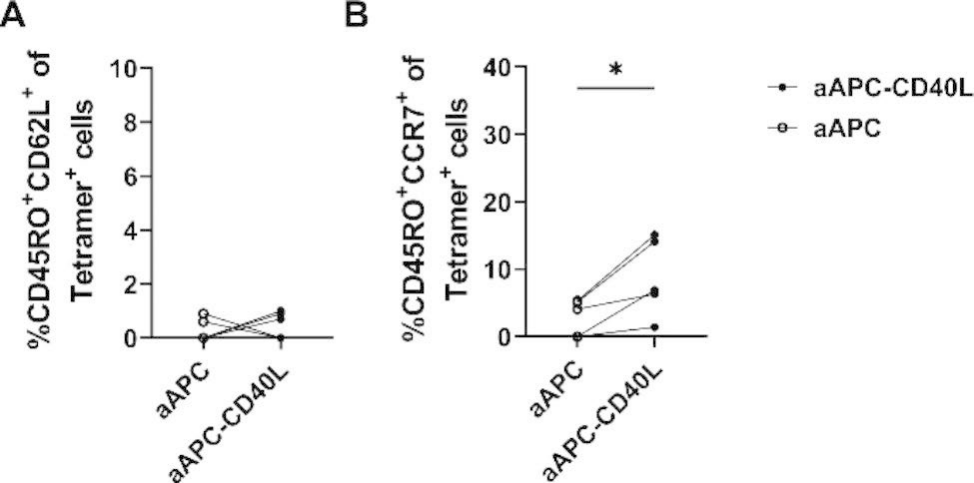
Effect of CD40L on the memory phenotype of CD8^+^ T cells specific for CMV pp65 antigen. Sorted CD8^+^ T cells were weekly stimulated with irradiated aAPC or aAPC-CD40L loaded with HLA-A*02:01 restricted peptides of pp65 in the presence of IL-2 (10U/mL). After two rounds of stimulation, flow cytometry analysis was done on day 12. (**A, B**) Effect of CD40L on memory phenotype of CD8^+^ T cells specific for pp65 antigens was analyzed by comparing the CD45RO^+^CD62L^+^ proportion of tetramer positive cells and the CD45RO^+^CCR7^+^ proportion of tetramer positive cells in aAPC stimulated group to aAPC-CD40L stimulated group on day 12 (n = 8). Wilcoxon paired rank test one tailed, *p < 0.05

To verify the effect of CD40L on the memory phenotype of pp65-specific CD8^+^ T cells, CD45RO^+^CD62L^+^ population and CD45RO^+^CCR7^+^ population among tetramer positive CD8^+^ T cells were analyzed by flow cytometry. The CD45RO^+^CD62L^+^ population among tetramer positive CD8^+^ T cells showed no difference between aAPC-CD40L stimulated group and aAPC stimulated group (Fig. 5A). However, CD45RO^+^CCR7^+^ population among pp65 tetramer positive CD8^+^ T cells was significantly higher in aAPC-CD40L stimulated group compared to aAPC stimulated group (8.8% ± 5.8% versus 2.9% ± 2.7%, P < 0.05) (Fig. 5B). This data suggests the effect of CD40L on memory generation of pp65-stimulated CD8^+^ T cells.

## Discussion

Previous studies demonstrated the importance of CD40L on the proliferation and generation of memory CD8^+^ T cells [5–9] but whether the effect is delivered to CD8^+^ T cells directly from CD4^+^ T cells or indirectly through APC has not been clearly identified especially in human peripheral T cells.

In this study, the possibility of direct CD40L effect was demonstrated by analyzing the expression of CD40 on sorted CD8^+^ T cells. Previous study reported transient expression of CD40 on CD8^+^ T cells after activation in mice [9] and our result showed that the same is true for human CD8^+^ T cells. Also, increased number of CD8^+^ T cells stimulated with aAPC-CD40L adds evidence to the proliferative effect of CD40L on T cells [13, 14]. Increased CD8^+^ T cell number of T_CM_ subset mostly attributed to the increase of total CD8^+^ T cell number (Fig. 2C). In pp65-specific CD8^+^ T cells, fold expansions of CD8^+^ T cells were also significantly higher in aAPC-CD40L stimulated group (Fig. 4A). As some other studies have shown, the CD40L expression on CD8^+^ T cells was also increased after stimulation (Additional File Fig. 1) [15, 16]. This suggests that the interaction between CD8^+^ T cells through CD40-CD40L might be possible and this could be responsible for the modest effect of CD40L expressed on aAPC-CD40L. However, the factor which derives the expression of CD40 and CD40L on CD8^+^ T cells should be further studied.

IFN-γ spot numbers (Fig. 4B) and the percentage of tetramer positive CD8^+^ T cell (Fig. 5A) were statistically not significant. These results suggest that CD40L increased the number of both antigen specific CD8^+^ T cell as well as non-antigen specific CD8^+^ T cells, namely the bystander CD8^+^ T cells. Previously, the effect of CD40L on the proliferation of bystander CD8^+^ T cells also has been reported [17, 18]. Since we used sorted CD8^+^ T cells without dendritic cells or B cells, our results show that CD40L has a direct effect on the proliferation of bystander CD8^+^ T cells in addition to the proliferation of antigen-specific CD8^+^ T cells. Also, the increased number of bystander CD8^+^ T cells might be due to the cytokines secreted from activated antigen-specific cells [19]. However, whether these bystander CD8^+^ T cells express CD40 on their surfaces or which factors are involved in the upregulation of CD40 has not been evaluated yet. Some studies have reported that IFN-γ upregulates CD40 expression on various cell types including macrophages, microglia, thymic epithelial cells, smooth muscle cells and endothelial cells [20–23]. Therefore, further study regarding the CD40 expression on bystander CD8^+^ T cells is needed.

Contrasting results have been reported on the effect of CD40L in the generation of memory CD8^+^ T cells [5, 9, 24, 25]. Some studies reported similar levels of secondary responses of CD8^+^ T cells and stable level of memory CTL precursors in the absence of CD40 signals to CD8^+^ T cells [25]. On the contrary, direct signaling of CD40L to CD40 on CD8^+^ T cells has been shown to be fundamental for CD8^+^ T cell memory generation [9]. In addition, whereas primary expansion of CD8^+^ T cells were similar with or without CD4^+^ T cell help, secondary expansion was limited when CD8^+^ T cells were primed without CD4^+^ T cell help [11]. Adding evidence to these previous reports suggesting the effect of CD40 signal on the memory generation, our results showed increase of CD45RO^+^CD62L^+^ CD8^+^ T cell population in T_total,_ T_N,_ and T_CM_ subsets when stimulated with aAPC-CD40L although the magnitude in T_N_ was small compared to that of in T_CM_ subsets. However, CD45RO^+^CD62L^+^ CD8^+^ T cell population level was similar when stimulated with aAPC compared to that when stimulated with aAPC-CD40L in T_EM_ and T_E_ subsets. Since T_E_ and T_EM_ cells are previously stimulated and differentiated cells, additional CD40L stimulation seems to have minimal additional effect.

In antigen specific stimulation, only the CD45RO^+^CCR7^+^ population of pp65 tetramer^+^ cells were higher in aAPC-CD40L stimulated group compared to that of aAPC stimulated group. pp65 can be thought as repeated antigen challenge since previous infections of CMV is common among population [26]. Because the exact distribution of memory subsets of each antigen specific CD8^+^ T cells was not examined, the effect of CD40L on the memory generation of pp65-specific CD8^+^ T cells could not be further analyzed in detail.

In this study, cell surface molecule expressions such as CD45RO, CD62L and CCR7 were used to determine the memory differentiation status of CD8^+^ T cells. These phenotypic markers are widely recognized as the parameters of T cell differentiation status [27, 28]. However, since T cells showing different characteristics with similar phenotype such as memory T cells with a naive phenotype has been discovered, the degree of differentiation cannot be absolutely determined only by the expression of surface markers [29–31]. Thus, additional evaluation methods such as analysis of proliferation, IFN-γ secretion and cytolytic effect after second stimulation may be combined to evaluate the memory differentiation status of CD8^+^ T cells.

This study has several limitations that should be considered. First, this study has been carried with a small size of sample. Studies with large sample size is further needed. Second, although the increased number of aAPC-CD40L stimulated CD8^+^ T cells seems prominent compared to that of aAPC stimulated CD8^+^ T cells, it is unclear whether this is due to increased proliferation or enhanced survival of CD8^+^ T cells. The exact effect of CD40L on CD8^+^ T cells should be evaluated by analyzing cell proliferation and survival. Finally, additional study which could evaluate the contribution of CD40-CD40L interaction within CD8^+^ T cells is needed since CD8^+^ T cells also express CD40L upon activation.

## Conclusions

In conclusion, our results suggest that CD40L has an effect on the increased number of CD8^+^ T cells through CD40 expressed on activated CD8^+^ T cells and has influence on memory CD8^+^ T cell generation. However, complex crosstalk between CD4^+^ T cells, antigen presenting cells and CD8^+^ T cells might nevertheless exist despite the ability of CD8^+^ T cells to receive direct signaling through CD40. Our results may provide a new perspective of the effect of CD40L on human peripheral CD8^+^ T cells, which differ between the memory differentiation status of CD8^+^ T cells. In future studies, functional aspects such as the cytolytic effect of the CD40L stimulated CD8^+^ T cells and the underlying mechanism of CD40 signal effect on CD8^+^ T cells should be evaluated.

## Materials and methods

### Cells

The use of human PBMC was approved by Institutional Review Board of the Catholic University of Korea (MC14TNSI0119) and written informed consent was obtained from participants. PBMCs were obtained from eight healthy donors, four of whom were male and four of whom were female. The median age was 31 years, with a range of 25 to 36 years. PBMCs were collected using Ficoll-Hypaque (GE Healthcare, Pittsburgh, PA, USA) and were preserved in liquid nitrogen. After thawing, PBMCs were stained with antibody and were sorted by flow cytometry (MoFlo Astrios flow cytometer, Beckman Coulter, Indianapolis, IN, USA). HLA typing was done by the Catholic Hematopoietic Stem Cell Bank (Seoul, Korea).

### Establishment of aAPCs

Establishment of aAPCs were done as previously reported [12]. In brief, costimulatory molecules CD32, CD83, 4-1BBL, CD80, HLA-A*02:01 and CD40L were cloned into the pCDH lentivirus vector (#CD523A-1;SBI, Palo Alto, CA, USA). Lentivirus production was done by cotransfecting cloned 10 µg of pCDH plasmid and 5 µg of lentivirus packaging plasmids pMD2.G and psPAX2 to HEK293 cells with lipofectamine (Invitrogen, Carlsbad, CA, USA). Lentiviral supernatant was added with 8 µg/mL of polybrene to K562 cells for transduction. After 2 days, costimulatory molecule and HLA expression was analyzed by flow cytometry (FACS Canto II, BD Biosciences, San Diego, CA, USA). Stable cell line which highly expressed HLA and costimulatory molecules was selected by sorting with flow cytometer (MoFlo Astrios flow cytometer, Beckman Coulter, Indianapolis, IN, USA).

### Stimulation of CD8^+^ T cells

For antigen non-specific CD8^+^ T cell stimulation, CD8^+^ T cells from PBMC were isolated and was further sorted by the expression level of CD62L and CD45RO by flow cytometer (MoFlo Astrios flow cytometer, Beckman Coulter, Indianapolis, IN, USA). Sorted or total CD8^+^ T cells were weekly stimulated with anti-CD3 loaded irradiated aAPC or aAPC-CD40L in the presence of 10U/mL interleukin-2 (IL-2) (Proleukin, Emeryville, CA). pp65-specific CD8^+^ T cells were generated from PBMC of HLA-A*02:01 donor using CMV pp65(495–503) peptide (JPT Peptide Technologies, Berlin, Germany) pulsed aAPC or aAPC-CD40L as stimulator cells. Sorted CD8^+^ T cells were weekly stimulated with irradiated stimulator cells in the presence of 10U/mL interleukin-2 (IL-2) (Proleukin, Emeryville, CA). After two rounds of stimulation, cells were harvested and analyzed for proliferation, pp65-tetramer positive population, memory differentiation and IFN-γ secretion.

### IFN-g ELISPOT Assay

For ELISPOT assays, BD ELISPOT assay kit was used according to the manufacturer’s instructions. Sorted CD8^+^ T cells were weekly stimulated with irradiated aAPC or aAPC-CD40L loaded with HLA-A*02:01 restricted peptides of pp65 in the presence of IL-2(10U/mL). After two rounds of stimulation, 2.5 × 10^6^ cells were serially diluted in complete RPMI and 125µL of each concentration was transferred to in an ELISPOT plate (BD Biosciences, San Diego, CA). Cells were co-cultured with 2.5 × 10^4^ pp65-pulsed T2 cells for 20 h at 37℃ in 5% CO2. Spot number was counted by AID-ELISPOT Reader System (AID Diagnostika GmbH, Straassberg, Germany).

### Flow cytometry

Cells for flow cytometry analysis were harvested and stained with fluorescently labeled antibodies for 30 min at 4℃ in dark. Following antibodies were used: anti-CD80 (L307.4; BD Biosciences, San Diego, CA), anti-CD32 (FLI8.26; BD Biosciences), anti CD83 (HB15e; Biolegend, San Diego, CA), anti-CD137L (5F4; Biolegend), anti-CD3 (OKT3, Biolegend), anti-CD8 (RPA-T8, Biolegend), anti-CD45RO (UCHL1, Biolegend), anti-CCR7 (G043H7, Biolegend), and anti-CD62L (DREG-26, BD Bioscience). pp65 Tetramer (Proimmune, Oxford, MC, UK) was used for tetramer staining and cells were analyzed with Canto (BD Biosciences). For analysis, lymphocyte population was gated based on FSC and SSC and further gating of CD8^+^ T cells were done by gating CD3 and CD8 double positive cells. Gating of memory differentiation was done either with CD45RO and CD62L or with CD45RO and CCR7.

### Statistical analysis

For statistical analysis and visualization, Prism 9.0 software (GraphPad, SanDiego, CA, USA) was used. Statistical significance was determined by paired t-test. P < 0.05 was considered significant.

## Electronic supplementary material

Below is the link to the electronic supplementary material.

Additional File 1: CD8^+^ T cells transiently express CD40L after stimulation

## Data Availability

The datasets used and/or analysed during the current study are available from the corresponding author on reasonable request.

## Abbreviations

APC: Antigen presenting cells
aAPCs: Artificial antigen presenting cells
TNF: Tumor necrosis factor
CTL: Cytotoxic T lymphocyte
HCMV: Human cytomegalovirus
T_N_: Naïve T cell
T_CM_: Central memory T cell
T_EM_: Effector memory T cell
T_E_: Effector T cell
PBMC: Peripheral blood mononuclear cells
IL-2: Interleukin-2
