# Supplementary material for: CD40 ligand stimulation affects the number and memory phenotypes of human peripheral CD8+ T cells
Source: BMC Immunol. 2023 Jun 30;24:15. doi: 10.1186/s12865-023-00547-2 (PMC10311846; doi:10.1186/s12865-023-00547-2)
Supplement: Supplementary file 1 — Additional File 1: CD8+ T cells transiently express CD40L after stimulation [file 12865_2023_547_MOESM1_ESM.docx]

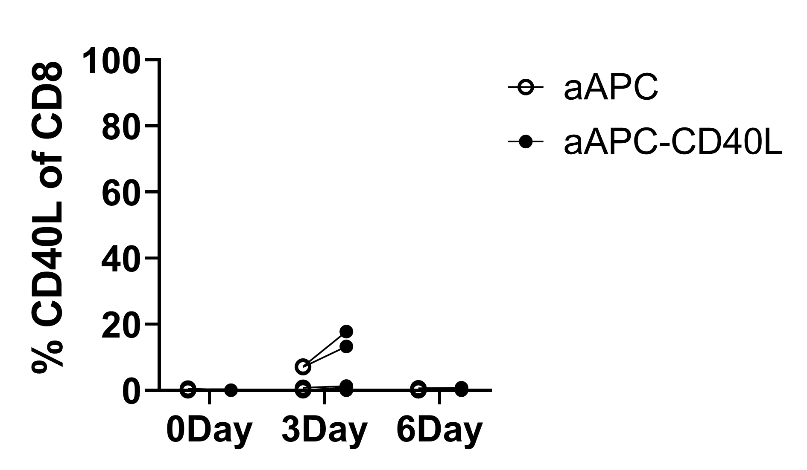


**Supplementary figure 1. CD8^+^ T cells transiently express CD40L after stimulation.** CD40L expression on human peripheral blood CD8^+^ T cells after stimulation with aAPC or aAPC-CD40L. CD8^+^ T cells were isolated from five healthy donor PBMCs by magnetic sorting and were stimulated with anti-CD3 loaded aAPC/aAPC-CD40L in the presence of IL-2 (10U/mL). Expression of CD40L on CD8^+^ T cells were analyzed on 0, 3 and 6 days after stimulation by flow cytometry. Representative FACS data and data from 5 donors on day 0, 3 and 6 are shown as dot plot.
